# Supplementary material for: A Longitudinal Study on Trajectories of Night Work and Sickness Absence among Hospital Employees
Source: Int J Environ Res Public Health. 2022 Jul 3;19(13):8168. doi: 10.3390/ijerph19138168 (PMC9265793; doi:10.3390/ijerph19138168)
Supplement: Supplementary file 1 [file ijerph-19-08168-s001.zip › ijerph-1803610-supplementary.pdf]

Supplemental material

Supplemental Table S1. Mean frequency of night shifts from 2008 to 2019 in trajectory groups in samples 1, 2, and 3.

	**Number of night shifts in Sample 1**											
	**2008**	**2009**	**2010**	**2011**	**2012**	**2013**	**2014**	**2015**	**2016**	**2017**	**2018**	**2019**
**Group**	**mean**	**mean**	**mean**	**mean**	**mean**	**mean**	**mean**	**mean**	**mean**	**mean**	**mean**	**mean**
"Group 1: stably no night shifts"	0.3	0.1	0.1	0.1	0.1	0.1	0.0	0.1	0.1	0.1	0.1	0.1
"Group 2: stably moderate number of night shifts"	33.1	33.1	34.1	34.7	34.3	34.7	35.3	35.8	36.2	35.0	35.9	33.9
"Group 3: stably high night shifts"	72.5	74.1	74.7	76.5	72.5	73.8	74.5	72.2	75.4	72.4	70.1	66.4
"Group 4: moderate and decreasing night shifts"	27.7	26.0	22.4	18.9	14.6	10.8	7.7	5.1	2.5	1.5	1.0	1.0
"Group 5: low and increasing night shifts"	6.9	5.6	5.5	5.4	6.2	8.0	11.8	14.1	15.5	14.7	19.8	20.9
	**Number of night shifts in Sample 2**											
"Group 1: stably no night shifts"	0.1	0.1	0.1	0.1	0.0	0.0	0.0	0.0	0.0	0.0	0.0	0.0
"Group 2: moderate and decreasing night shifts"	53.5	41.1	42.7	35.2	34.0	36.4	35.0	33.5	28.3	28.8	30.0	23.0
"Group 3: low and decreasing night shifts"	18.0	9.4	10.0	8.9	7.0	7.5	7.6	7.4	6.0	6.7	6.0	5.4
	**Number of night shifts in Sample 3**											
"Group 1: stably no night shifts"	0.0	0.0	0.0	0.0	0.0	0.0	0.1	0.0	0.0	0.0	0.0	0.0
"Group 2: moderate and fluctuating night shifts"	42.0	31.1	28.6	34.4	30.4	28.6	23.2	27.0	26.9	25.4	26.1	35.1
"Group 3: stably low night shifts"	10.7	7.6	6.2	6.5	7.6	6.7	7.2	6.7	5.6	5.6	6.4	7.4
